# Novel Strategy in the Detection of Adverse Cutaneous Drug Reactions: A Case Series Study

**DOI:** 10.3390/diagnostics14060575

**Published:** 2024-03-07

**Authors:** Erika-Gyöngyi Bán, Patrick Lechsner, Eszter-Anna Dho-Nagy, Maria-Antonia Balan, István Major-Szakács, Attila Brassai, Zsuzsánna Simon-Szabó, Corina Ureche

**Affiliations:** 1 Faculty of Medicine in English, Preclinical Research Laboratory, “G.E.Palade” University of Medicine, Pharmacy, Science and Technology Targu Mures (UMFST), 540142 Targu Mures, Romania; erika.ban@umfst.ro (E.-G.B.);; 2 Independent Researcher, 540320 Targu Mures, Romania; 3 Faculty of Medicine, “G.E.Palade” University of Medicine, Pharmacy, Science and Technology Targu Mures (UMFST), 540142 Targu Mures, Romania

**Keywords:** side effect, ultrasound, pharmacotherapy

## Abstract

With multimorbidity on the rise, adverse cutaneous drug reactions are becoming a daily challenge in clinical practice. The objective evaluation of the skin lesion is crucial but hardly realized due to missing technology and guidelines. In this study, the novel Dermus SkinScanner-U, an optically guided high-frequency ultrasound imaging device, was evaluated regarding its comparability with the Dermatology Life Quality Index (DLQI) and the pharmacological analysis of the patients’ drug therapy. A total of 40 adult patients were evaluated, all with chronic medication use and skin lesions that led to non-compliance toward the pharmacotherapy. With the ongoing aim of further improving the methodology, the first results, with two detailed patient cases, are presented here. It was concluded that in the cases evaluated, there was a significant correlation between the characteristics of the lesions observed on the optical and ultrasound image, the DLQI score, and the pharmacological analysis. The next steps include increasing the scale of the study to ultimately develop a quality-assured methodology for the correct diagnosis of skin-related adverse drug reactions and to prepare a database with the most frequently observed events.

## 1. Introduction

As a consequence of the demographic shift towards an aging population in Europe, the healthcare system is confronted with an increasing number of chronic diseases and multimorbidity. These are most commonly treated with chronic drug treatment raising concerns of adverse drug reactions (ADRs) especially due to the increasing number of co-administered medications [1]. Chronic diseases, often necessitating the use of multiple medications, account for over 90% of annual healthcare expenditures in the US, amounting to a staggering 4.1 trillion USD [2]. Consequently, the widespread utilization of pharmaceutical drugs contributes to approximately 1.3 million emergency room visits annually due to adverse drug reactions (ADRs) [3]. While it is estimated that at least 3.5 billion USD are allocated to treating adverse drug reactions (ADRs) each year, accurately quantifying the costs specifically attributed to ADRs remains challenging [4,5].

The role and aim of clinical pharmacology and clinical pharmacologists were defined as the individualization of pharmacological therapies. However, in the last years, it became obvious that there is a difference between the efficacy and the safety of treatments during a controlled clinical trial and normal everyday practice [6].

Recently, we as clinical pharmacologists are facing the situation where the continuous monitoring of drug effects on the organism after its regulatory approval is considered an integral part of the drug evaluation. This formulates new requests and new tasks, like drug utilization studies and the behavior of the drug in everyday practice, namely the drug–individual–society interaction [7].

**Figure 1:**
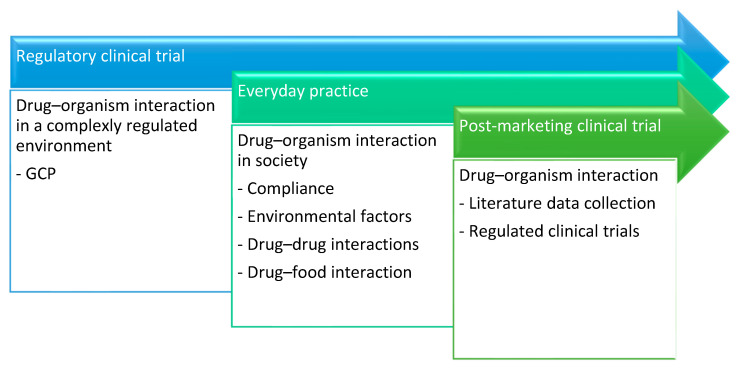
The role and duties of the clinical pharmacologist in the different therapeutic routines during the lifecycle of the drug.

**Figure 2:**
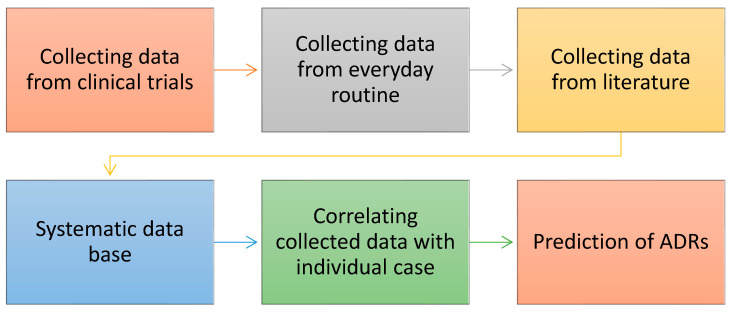
The clinical pharmacological process for the prediction of ADRs.

An additional objective within the specialty of clinical pharmacology is to assume an active role in the surveillance, identification, and appropriate treatment of adverse drug reactions (ADRs) [8]. Despite the efforts of the pharmacovigilance systems, several ADRs remain unnoticed and/or not identified as drug-induced side effects [9]. Enhancing the likelihood of identifying adverse drug reactions (ADRs) necessitates closing the disparity between clinical trial data and the inherent variability of non-controlled treatment parameters. Therefore, it is imperative to adopt data collection and systematic review methodologies tailored to “real-world therapeutics”, derived from routine, everyday practices [10,11]. This approach can potentially enhance the predictability of adverse drug reactions (ADRs) while concurrently reducing their severity. A summary of the role and duties of a clinical pharmacologist can be found in Figure 1 and Figure 2 elicits the process used for prediction of ADRs.

Drug-induced skin reactions or adverse cutaneous drug reactions (ACDRs) account for up to 30% of these adverse events; these reactions are called toxidermia [12]. According to the World Health Organization, 2% of ACDRs are classified as serious adverse drug reactions [13]. The exact mechanism of drug-induced dermatological responses is not completely understood, but the underlying patho-mechanism is frequently of immune, inflammatory, or allergic nature [14]. 

ACDRs can be subclassified into acute or chronic and localized or systemic reactions. Diagnosing the condition poses a challenge, necessitating the meticulous analysis of the patients’ pharmacotherapy alongside a comprehensive understanding of other potential causative factors. [15]. Adverse drug reactions (ADRs) frequently result in patient non-compliance with their prescribed medication regimen, particularly when the causative factor remains unrecognized until later stages [16]. In the case of a suspected ACDR, even if symptoms are mild, the early recognition and identification of the underlying pharmacotherapeutic agent are key to alleviate the dermatological symptoms and ensure patient compliance [17]. Diagnostic criteria of ACDRs are listed in Table 1.

In Romania, the integration of clinical pharmacologists’ responsibilities into clinical practice is currently underway, driven by the annual increase in observed adverse drug reactions (ADRs). We believe that exploring avenues that support the clinical pharmacological evaluation of individual patients is highly beneficial. Documenting these patients’ assessments aims to provide solid evidence within the aforementioned database.

The primary goal of the clinical pharmacologist is to diminish the need for medical care, hospitalization duration, and mortality, all resulting from ADRs. Achieving this goal hinges on promoting the rational use of medications, which is contingent upon the comprehensive consideration of data spanning the entire lifecycle of medicines [18,19].

According to the latest Pharma & Hospital Report, Cegedim Customer Information data [20,21], Romania demonstrates a significant consumption of medications. The study reveals a 7.2% increase in medication usage in 2022, with prescription drugs from pharmacies constituting 57% of the total, while over-the-counter products accounted for 43%. The analysis of drug groups reveals that medications for the cardiovascular system (25.5%), digestive system (25.5%), central nervous system (18.5%), and respiratory system (14.7%) are the most commonly utilized. The top 10 medications used in Romania include metamizole, ibuprofen, aspirin in 125 mg and 500 mg doses, amoxicillin, diosmin, indapamide, and enalapril. Notably, each of these medications has the potential to induce skin-related adverse drug reactions [22].

Drawing upon the worldwide and local literature as well as our own database, it is evident that adverse drug reactions (ADRs) affecting the skin represent a significant concern for clinical pharmacologists [23,24]. 

## 2. Objectives

The primary objective of the present study was to try to establish a proposed algorithm for the clinical pharmacological evaluation of skin related ADRs.

During this study, we aimed to objectively evaluate the usefulness of images obtained by a novel skin imaging device, the Dermus SkinScanner-U, in the observation and classification of ACDRs. 

It was hypothesized that the Dermus SkinScanner-U optically guided high-frequency ultrasound imaging device could be successfully used by clinical pharmacologists to objectively observe skin changes in the context of ACDRs. The secondary objective was to analyze possible correlations between objective clinical investigations performed using the Dermus SkinScanner-U, Dermatology Life Quality Index (DLQI), and the clinical pharmacological evaluation of the drug therapy, in adult patients with skin-related complaints.

## 3. Materials and Methods

**Figure 3:**
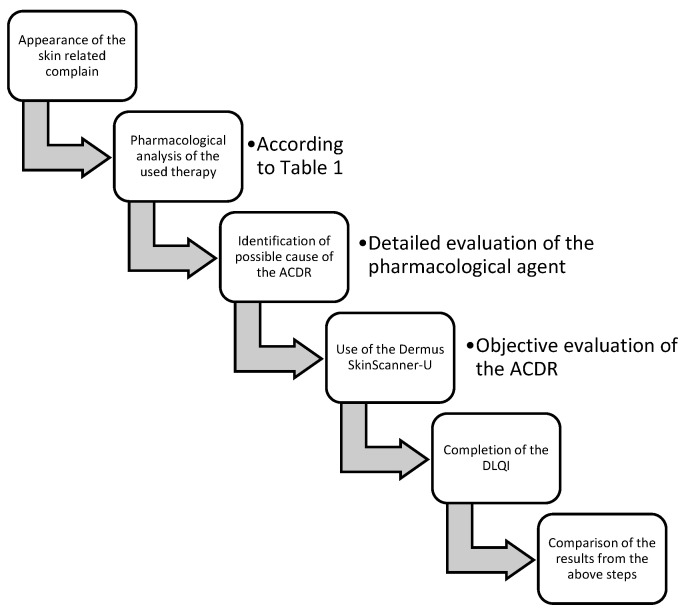
Methodology of the study.

This project was performed as a prospective, non-interventional pharmacological study in parallel with the dermatological non-interventional evaluation of skin lesions with the Dermus SkinScanner-U and the completion of the Dermatology Life Quality Index. Figure 3 describes the methodology used. This study was conducted according to the ethical approval of the “G.E.Palade” UMPhST’s Ethical Committee Nr. 1874/29.09.2022.

(1) The clinical pharmacological assessment of the drug regimen was performed using a checklist (Table 2) to identify possible drug causes for the assumed ACDR.

(2) “The Dermus SkinScanner is a wireless, compact (handheld), portable multimodal optical and ultrasound imaging device that is developed specifically for skin imaging by Dermus Ltd. (Budapest, Hungary)” [25]. In comparison to other portable ultrasound imaging devices, the Dermus SkinScanner uses optical guidance for the enhancement of precise positioning and the repeatability of the recordings, and the device is easy to use by non-radiology specialists. The Dermus SkinScanner-U, the model used in this study, is a novel, research-use version of the above-mentioned Dermus SkinScanner device with an enhanced user experience, the possibility of taking multi-frame recordings, and more precise scanning.

The Dermus device provides an innovative solution for skin diagnostics. It employs high-frequency ultrasound waves to image subsurface structures. These waves, generated by the device, interact with the skin’s structural elements, scattering and reflecting off any inhomogeneities. The reflected waves are then received and processed by the device. Utilizing a one-element transducer, which is moved along a linear stage by a stepper motor, the device converts the received data into two-dimensional images, accounting for both forward and backward movements. The exact imaging specification can be seen in Table 3. Optical imaging assists in guiding the ultrasound imaging process, ensuring accurate capture. The spatial position of the ultrasound image cross-section is denoted by a red line overlaid on the optical image. In order to provide higher contrast and facilitate the detection of skin structures, the ultrasound image employs a color-scale instead of a grayscale representation. A sequence of corresponding optical–ultrasound image pairs can be recorded, with the option to save selected pairs or the entire set for storage and further analysis. While it is currently for research use only, it offers several features, like AI-Assisted Web App (version R2401) that works in conjunction with the CE-certified hardware device; Skin Lesion Diagnostics, the device that creates images to measure and analyze various skin parameters, assisting in the diagnosis and treatment of skin conditions; Patient Management, where Dermus enables anonymized data collection for patient management; and Smart Image Annotation and Filters, to annotate images, apply filters, and adjust settings. This technology provides help not only for dermatologists but also for other medical specialists. 

The Dermus SkinScanner-U examinations were conducted by a team comprising a clinical pharmacology specialist, a resident doctor specializing in clinical pharmacology, and a sixth-year medical student who also served as a teaching assistant in the pharmacology department. Prior to the examinations, the examiners received training from the developer and application engineer responsible for the device. It is important to note that each examination was conducted collaboratively by the entire research team rather than individually by one member to ensure maximum reliability. Multiple images and scans were taken during the examination of each patient. The lesions were analyzed using the tools provided by the ‘SkinAid’ application interface (by Dermus Ltd., Budapest, Hungary). The ‘SkinAid’ application offers easy evaluation of lesions for clinical pharmacology specialists or general practitioners.

(3) The third component of the ACDR evaluation was the completion of the Dermatology Life Quality Index (DLQI) by the patients, with assistance when needed.

After the completion of all the above-mentioned steps, the correlation between pharmacological assessment, Dermus SkinScanner-U imaging analysis results, and the DLQI score was checked.

## 4. Results

In the current study, 40 adult patients were evaluated, all with chronic medication use and skin lesions that led to non-compliance toward the pharmacotherapy. With an ongoing commitment to refining our methodology, we present the initial outcomes featuring detailed analyses of three patient cases. All three exemplify that ACDRs can occur suddenly, even after years of medication use, and the difficulty of not only diagnosing ACDRs but finding the causative agent.

**Case 1:**

**Figure 4:**
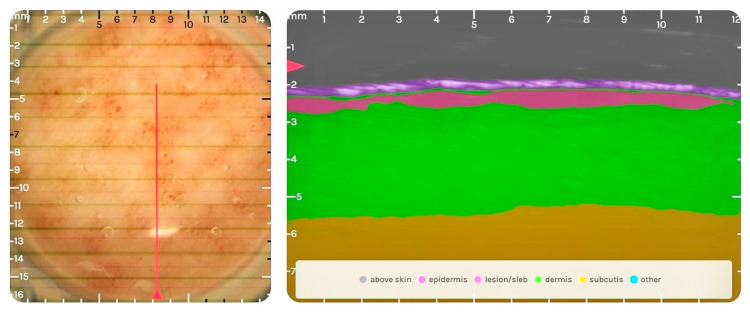
Optical (**left**) and ultrasound image (**right**) of the lesion: The subepidermal low-echogenic band (SLEB) can be seen on the ultrasound image colored in pink, between the epidermis and dermis. The echogenicity of the lesion also reveals dermatitis: the increase in the echogenicity of the epidermis and the slightly decreased echogenicity of the dermis due to the decreased hydration levels.

A 57-year-old male patient presented with local erythema of the left lower limb without urticaria, pain, or pruritus. Relevant personal and medical history of the patient includes essential arterial hypertension under pharmacological treatment for 6 years and skin-related complaints for 4 years. The essential arterial hypertension was diagnosed during a routine medical check-up by the general physician and therapy was started with the retard formulation calcium-channel blocker (CCB) nifedipine and a thiazide-like diuretic, indapamide. Pharmacotherapy was considered efficient due to normal blood pressure readings and was continued without modification. The patient started to experience dermatological symptoms at the level of the left lower limb after the first year of therapy. It was diagnosed by the general practitioner as a local allergic reaction for which local therapy with antihistamine ointment was initiated without any success. The patient stopped the local antiallergic therapy and the skin-related lesion worsened after the dose of the CCB was eventually increased from the daily dose of 40 mg to 80 mg. Finally, this led to non-compliance with the patient discontinuing the complete antihypertensive regimen. Evaluation in the context of this study revealed an increased area of redness, itching, dull pain, and exfoliation at the center of the lesion. The DLQI score was 13 at the time of evaluation. The lesion was analyzed with the Dermus SkinScanner-U as seen in Figure 4.

The patient was on CCB monotherapy for the past 6 years with nifedipine oral tablets of 80 mg taken daily. According to the pharmacological characteristics of this active substance, skin-related side effects can appear on a rare, non-specific basis with an increase in incidence during long-term and high-dose usage, both factors being present here. Considering the above-mentioned information, the use of nifedipine tablets can be considered a pharmacological cause of the patient’s dermatological problem, though only provable with the discontinuation of the medication and the disappearance of the ACDR.

The DLQI score of the patient was 13 (11–20 meaning a very large effect on the patient’s life) at the time of the evaluation, but the patient clearly states a large, negative effect on his life for the last 4 years. The evaluation of the lesion with the Dermus SkinScanner-U and analysis with the ‘SkinAid’ App revealed a dermatological lesion corresponding to dermatitis, the presence of a subepidermal low-echogenic band (SLEB)—characteristic for skin inflammations [12,13]—with a thickness of 0.55–0.87 mm on the ultrasound image (Figure 4) and clinical signs of mild/moderate erythema and erosions. The replacement of nifedipine as an antihypertensive and the monitoring of the ACDR was recommended.

**Case 2:**

A 72-year-old male patient presented with a feeling of pins and needles and itching of the skin associated with redness and hyperpigmentation. Relevant personal and medical history includes recurrent depressive disorder for 28 years, hypertension for 15 years, gastro-esophageal reflux disease for 5 years, and sleep disorder for 15 years, altogether treated with more than five drugs concomitantly. The drug regimen of the last 5 years included the continuous use of a serotonin–norepinephrine reuptake inhibitor (SNRI) in varying doses, an angiotensin-converting enzyme inhibitor (ACE-I), a calcium channel blocker (CCB), a thiazide diuretic, an anticonvulsant, and, periodically, a proton-pump inhibitor (PPI). As the dermatological symptoms worsened, the compliance decreased, and the patient stopped taking the medication(s) irregularly. On a non-regular basis. The clinical pharmacological analysis of the drug therapy revealed four moderate and two mild drug–drug interactions as well as multiple drugs with a side effect profile explaining a possible ACDR. 

**Figure 5:**
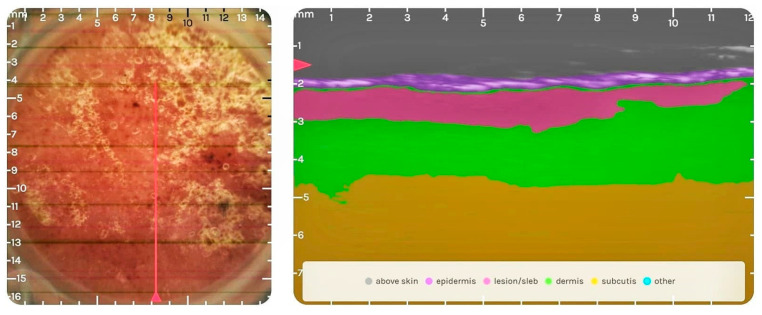
Optical (**left**) and ultrasound (**right**) image of the lesion: The optical image reveals the presence of abrasion, desquamation, and severe dryness of the skin. The analysis of the ultrasound image with the SkinAid App underlines that the thickness of a subepidermal low-echogenic band (SLEB) is in correlation with the severity of the inflammation as well as the thickness of the dermis. The dry skin and erythema with erosions are visible on the optical image and correlate with the presence of SLEB, the decreased thickness of the dermis.

The SNRI venlafaxine is an active substance with known skin-related side effects, especially when used chronically. ACEI medications are known to induce bradykinin-mediated allergic side effects, including dermatological changes. Gabapentin, an anticonvulsant, has a moderate incidence in causing dermatological adverse effects. The CCB amlodipine and the thiazide diuretic used by the patient are potentially involved in the appearance of skin-related side effects but very rarely (non-reported frequency). The PPI was not used regularly and therefore would not explain the ACDR. The DLQI score of the patient at the time of examination was 9 (6–10 meaning a moderate effect on the patient’s life). Meanwhile, the Dermus SkinScanner-U optical and ultrasound images of the evaluated lesion (Figure 5) showed signs of a more severe inflammation than that of Case 1 above (larger SLEB thickness on the ultrasound image, and significant plaque on the optical image in Case 2). According to the personal anamnesis of the patient, we concluded that the depressive disorder had the potential to significantly influence the DLQI score. This was because he did not perform several relevant everyday activities referenced in the DLQI and therefore did not report the corresponding potential skin complaints (which would have increased the DLQI score). We treated this as a viable explanation for the objective evaluation showing a more severe ACDR than the DLQI score of this patient.

The exact, expected skin-related side effects according to the pharmacological characteristics of the above-listed drugs are shown in Table 4.

The non-compliance with the therapy led to decreased drug efficacy, and symptoms of the patient’s primary disease were worsening. The detailed history of the medication usage and the analysis of the drug regimen showed a possible correlation between subjective and objective complaints. The optical and ultrasound imaging performed with the Dermus SkinScanner-U revealed the presence of the shown lesion (Figure 5). The lesion is characteristic for abrasion, desquamation, and dry skin with the presence of SLEB on the ultrasound image. We concluded that the medication most likely to be the cause of the symptoms was gabapentin. Replacing the drug, or withdrawing it if the treatment is no longer required, and continuous monitoring were recommended.

**Case 3:**

A 33-year-old female patient presented herself with the following complaints: a periodically appearing rash and pruritus accompanied by local pain on various body regions. At the time of the examination, we observed very mild erythema on the right flank.

Relevant personal and medical history dates back four years and includes the following medical diagnoses: SARS-CoV-19 infection in 2020 with moderate symptomatology, which required hospitalization but no need for oxygen use at the time. Post-infection, the patient developed an anxiety disorder with insomnia for which she received pharmacological treatment for 6 months. The pharmacotherapy consisted of alprazolam and zolpidem tablets. Following six months of treatment, which was deemed ineffective, the patient opted to discontinue medication usage and has since been actively engaged in psychotherapy. In the last 3 years, the patient developed two or three urinary tract infections per year, and she suffered from bronchitis every winter. For each of the urinary tract infection episodes, as well as for the bronchitis, she received medical therapy. The medical therapy for both types of infectious illnesses consisted of a 7-day regimen of antibiotics, non-steroidal anti-inflammatory drugs, prokinetics, and a proton-pump inhibitor. An intriguing observation is that during each episode of infection, the patient consistently received the same antibiotic, amoxicillin, and the same NSAID, ibuprofen, albeit in varying doses. The prokinetics were different each time and the proton-pump inhibitor was initially omeprazole, switched afterwards to pantoprazole. Besides the above-listed medical therapies, the patient admitted that she uses birth control pills containing estrogen and progesterone as well as cranberry tea in aiming to prevent urinary tract infections. According to the patient’s records, she received various creams and ointments for her skin-related symptoms; however, none were used for more than two weeks due to their perceived inefficacy.

During this clinical pharmacological analysis, particular focus was placed on separately examining the medications administered during acute infectious periods and evaluating the birth control pill in terms of potential adverse drug reactions.

During the urinary tract infections and the bronchitis episodes, the patient used four medications concomitantly for seven days. The drug–drug interaction analysis revealed no significant interactions. Based on the individual analysis of the used medications, we observed a skin-related side effect profile for amoxicillin, for ibuprofen, and for the proton-pump inhibitor [27].

The contraceptive pill shows a drug–drug interaction with amoxicillin but without skin related symptomatology. The estrogen –progesterone-containing birth control pill utilized by the patient is known to potentially induce skin-related side effects. An overview of expected dermatological side effects of the patient’s medications can be found in Table 5.

The DLQI score of the patient at the time of examination was 14 (11–20 meaning a very large effect on the patient’s life). According to the patient’s statement, the score remained constant throughout the past three years.

**Figure 6:**
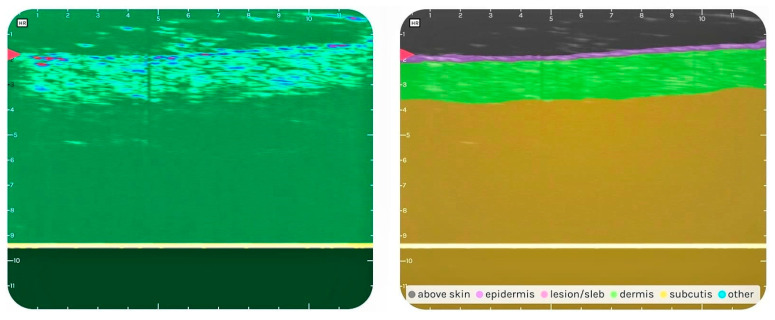
Ultrasound images of the lesion: Ultrasound image (**left**) and ultrasound image analyzed with SkinAid App (**right**). The ultrasound image reveals an absence of pathological alterations in the affected area. The appearance of the dermis, the subepidermal region, is without abnormal modifications except for a slight increase in the thickness of the dermis. Altered echogenicity cannot be observed.

The lesion was analyzed with the Dermus SkinScanner-U as seen in Figure 6.

According to the clinical pharmacological assessment of the patient’s treatment, taking into consideration the symptomatology and personal medical history, a diagnosis of atopic dermatitis and urticaria was considered plausible. The physical examination detected dermographism, which was sustained during the examination with the Dermus SkinScanner-U device, revealing the absence of pathological alterations in the subepidermal region characteristic of dermatitis.

According to the optical and ultrasound findings, the conclusion drawn was acquired dermographism. Through clinical pharmacological evaluation, it was determined that only one medication from the patient’s therapeutic regimen—amoxicillin—could potentially induce the onset of dermographism either during or after treatment.

## 5. Discussion

The three cases described above do not only show the methodology of the study but also how debilitating ACDRs can be for a patient. Furthermore, Case 1 exemplifies that even ‘simple’ monotherapy patients can experience ACDRs, while Case 2 demonstrates the difficulty of assessing polypragmasy patients. It also reveals how diagnosing and treating ACDRs require an objective evaluation to identify and remove possible causative factors or to rule out an ACDR in the first place. In this study, the objective evaluation of ACDRs was performed using the Dermus SkinScanner-U and the tools of the ‘SkinAid’ app. The diagnosis of ACDRs remains difficult with no gold standard established for the confirmation of the drug in question, especially in the case of polypragmasy. Important features to be taken into consideration are the time of drug exposure, the onset time of the reaction, the course of the reaction if the drug was withdrawn or not, and the characteristics of the skin lesion. The recurrence or eventual cross-reaction in the medical history can become relevant as well. Cutaneous drug reactions can become chronic when the etiological factors are present over a prolonged period or cease by themselves [28,29]. One specific characteristic to keep in mind is that the severity of the ACDR is not necessarily influenced by the dose of the medication. 

The manifestation of ACDRs and other skin-related pathologies can vary significantly based on their site of appearance. This variability has implications for diagnosis, management, and patient experience. When assessing a skin lesion, it is crucial to consider the localization and anatomical characteristics of the affected area. Lesions occurring on sun-exposed areas such as the face, neck, and arms are particularly delicate, prone to sun damage and aging. Skin cancers, including basal cell carcinoma, squamous cell carcinoma, and melanoma, are more commonly observed in these regions. On the extremities, it is important to distinguish between the palms, flexural areas, and nails. The skin of the palms exhibits unique patterns known as dermatoglyphics and is susceptible to conditions like palmoplantar psoriasis or warts. In contrast, flexural areas mainly present inflammatory skin diseases. The skin of the genitalia represents a distinct and sensitive area prone to infections, necessitating careful examination. Anatomical localization not only influences diagnosis and evaluation but also impacts pharmacotherapy. Considerations include the ease of applying topical medications to accessible areas and the potential impact of certain lesions and treatments on the patient’s appearance and quality of life. It is essential, however, to recognize that while the anatomical site of lesions is a significant aspect of evaluation, it should not be solely relied upon. Uncommon occurrences, such as melanoma occurring in non-sun-exposed areas, can still occur [30]. 

Dermatology stands out as one of the rare specialties where the target organ, the skin, is easily accessible for study, visualization, and biopsy. Biopsy remains the gold standard for diagnosing most skin lesions. However, recent years have witnessed significant advancements, granting clinicians and researchers the ability to non-invasively study the skin in real-time through high-frequency imaging devices. Consequently, ongoing technological progress offers promising prospects for diagnosing skin lesions in a non-invasive manner [31,32]. Ultrasonography was initially employed in dermatology for studying inflammatory skin disorders, particularly focusing on conditions like scleroderma. Presently, ultra-high- and high-frequency ultrasonography have expanded their utility to include oncologic, vascular, and a variety of inflammatory disorders, whether they are linked to medical therapies or not [31,33,34]. This technology not only aids in the diagnostic process but also enables follow-up examinations, the assessment of treatment responsiveness, and the early detection of changes. Table 6 shows an exemplary overview of disorders where an ultrasound is utilized and the corresponding characteristic changes observed in sonography.

Specifically, the Dermus SkinScanner-U holds significant potential for the early detection of skin tumors. This capability enables prompt intervention with minimally invasive treatments, leading to improved prognoses. A prior study demonstrated that the Optical Guidance High-Frequency Ultrasound (OG-HFUS) even surpassed the novel Melanoma-Specific Imaging (MSI) in accurately estimating Breslow thickness and diagnostic precision. Consequently, the Dermus SkinScanner-U emerges as a promising solution to enhance preoperative diagnosis, thereby diminishing the necessity for re-excision, and ultimately enhancing patient outcomes while simultaneously curbing healthcare expenditures [36].

According to literature data, skin-related side effects are highly prevalent and significantly impact patients’ quality of life. Dermatological research tools play a crucial role in comprehending drug properties, refining treatments, and enhancing patients’ well-being.

Clinical pharmacologists are responsible for reviewing patients’ medication regimens to ensure appropriateness, safety, and efficacy. This involves assessing potential drug interactions, contraindications, and adverse effects. Through collaboration with other healthcare professionals, clinical pharmacologists adjust drug doses or suggest alternative therapies. An essential aspect of a clinical pharmacologist’s role is investigating adverse drug reactions (ADRs) and providing recommendations for management. This includes offering expertise and educating patients about their medications, potential side effects, and adherence to the drug regimen. Clinical pharmacologists aim to bridge the gap between the efficacy and safety results observed in clinical trials and their applicability to everyday practice. When evaluating adverse drug reactions (ADRs), particularly skin-related side effects, the utilization of the Dermus SkinScanner-U device can significantly expedite the diagnostic process and offer a reliable method to exclude organic dermatologic lesions, thereby enhancing patient safety.

Several recent studies have been performed to identify the most probable causes of ACDRs among specific populations or hospital settings. It is often difficult for the general practitioner or clinical pharmacologist to correctly determine the severity of the complaint based on the subjective description of the patient or even based on the DLQI score. In the present pilot study, the aim was to check the possibility of correlation between the aspects of pharmacological analysis, the DLQI score, and the optically guided ultrasound imaging examination (performed with the Dermus SkinScanner-U). The investigation for ACDR confirmation always involves the analysis of the time from the initial exposure or re-exposure to the appearance of the skin lesion, the resolution of lesions after the discontinuation of the drug, the nature of the lesions, and a history of similar reactions to the suspected drug. Unfortunately, confirmation is frequently subjective, and a long-term objective follow-up is often not possible either, even though in the cases of multimorbid patients who require chronic concomitant use of multiple drugs, it would be extremely useful. With the algorithm presented in this study, this deficiency can be corrected.

While this study primarily focuses on the diagnostic phase of adverse cutaneous drug reactions (ACDRs), it is crucial to emphasize that in the management of ACDRs, the prompt withdrawal or gradual tapering of the suspected causative drug is paramount. Additionally, it is imperative to refrain from introducing additional dermatological treatments, as they may exacerbate the patient’s signs and symptoms or hinder objective assessment. 

A major limitation of the present study is the lack of the measurement of plasma levels of the drugs under investigation. Our study, conducted as a case series, reveals several limitations that warrant acknowledgment [37]. Due to the restricted amount of data, our study remains descriptive in nature, lacking generalizability. The primary aim of our investigation was to explore evidence regarding the utilization of the Dermus SkinScanner-U, a novel device distinguished by its unique feature among portable ultrasound imaging devices: optical guidance for precise positioning and recording repeatability. This feature renders the device particularly intriguing for non-dermatology specialists. Additionally, it is worth noting that our prospective clinical investigation encompassed all patients with impaired Dermatology Life Quality Index (DLQI) scores and multiple drug regimens. However, for the purpose of this paper, we have focused on presenting the most intriguing and noteworthy cases, which in itself can be viewed as a limitation of the study. While dermatological pathologies were ruled out through the evaluation of skin lesions using the Dermus SkinScanner-U device, it is important to note that our study lacks a comparison with a dermoscopy examination, which constitutes another limitation. As our study was of a clinical pharmacological nature, we refrained from conducting cutaneous biopsies or allergological testing. However, all patients were strongly advised to seek consultation with an allergology specialist for further examination.

## 6. Conclusions

This present pilot study aimed to evaluate medication–therapy-related skin lesions with the help of optical and ultrasound imaging in correlation with pharmacological analysis. Due to the subjectivity, skin-related complaints present some difficulty for dermatology specialists, but even more for doctors from other clinical specialties, including clinical pharmacology. 

According to our results, we can conclude that in the cases evaluated, there was a significant correlation between the characteristics of the lesions observed on the optical and ultrasound image, the DLQI score, and the pharmacological analysis. Based on these observations, we intend to expand the study on a broader scale to investigate the prevalence and incidence of ACDRs in our region. The goal is to develop a quality-assured methodology for the correct diagnosis of skin-related adverse drug reactions and to prepare a database documenting the most frequently observed events. 

## Figures and Tables

**Table 1:** Diagnostic criteria of ACDRs.

Manifestations that do not resemble the pharmacological action of the drug
Reactions are similar to those caused by another antigen
An induction period typically lasting 7–10 days after initial exposure to the drug
Reproduction of the reaction by cross-reacting chemical structures
Reproduction of the reaction by a minimal dose of the drug
Resolution of the reaction upon discontinuation of the drug
Occurrence of the reaction in a minority of patients receiving the drug

**Table 2:** Steps of clinical pharmacological assessment.

Evaluated Criteria	Yes	No	N/A
***Patient-related information***			
Demographic data - Age - Gender - Living and work environment			
Pregnancy/breast feeding			
***Patient’s medical conditions***			
Past medical history			
Current medical diagnosis/es			
***Patient’s drug therapies***			
Past medication record			
Current medication record—prescription			
Current medication record—non-prescription			
Current medication record—other			
Social drug use/abuse			
Allergies			
Adverse reaction/s			
***Current medication record assessment***			
Drug vs. indication			
Drug vs. dosage regimen			
Drug vs. outcome			
Drug vs. compliance			
Drug vs. adverse reaction			

N/A—not applicable.

**Table 3:** Imaging specifications of Dermus SkinScanner-U [26].

**Ultrasound frequency range**	20–40 MHz
**Ultrasound penetration depth**	Up to 10 mm
**Ultrasound field of view**	10 mm (depth) × 12 mm (lateral)
**Mechanical index (MI):**	<0.59 ± 13%
**Soft-tissue thermal index (TIS):**	<0.12 ± 27%
**Optical field of view**	12 mm × 12 mm
**Frame rate**	1+ Hz (for both optical and ultrasound)

**Table 4:** Expected dermatological side effects of the patient’s medications (Case 2).

**ACEI**	Common: Pruritus, rash Uncommon: Urticaria, angioedema, eczema
**Calcium Channel Blockers**	Common: Flushing Uncommon: Pruritus, rash
**SNRI**	Common: Pruritus, rash Uncommon: Acne, contact dermatitis, urticaria
**GABA analogue**	Common: Abrasion, dry skin, pruritus, rash Uncommon: Eczema, urticaria, pigmentation

**Table 5:** Expected dermatological side effects of the patient’s medications (Case 3).

**Amoxicillin**	***Incidence not known:*** Blistering, peeling, or loosening of the skin, hives, pale skin, pinpoint red spots on the skin, rash, redness, soreness, or itching skin, sores, welting, or blisters, tenderness of the skin, unusual bleeding or bruising, yellow skin ***Common*** (1% to 10%): Erythema, exanthema, rash ***Uncommon*** (0.1% to 1%): Urticaria, pruritus ***Very rare*** (less than 0.01%): Angioedema, hypersensitivity vasculitis ***Frequency not reported***: Erythematous maculopapular rashes, erythema multiforme, Stevens–Johnson syndrome, bullous dermatitis, exfoliative dermatitis, toxic epidermal necrolysis/Lyell’s syndrome, acute generalized exanthematous pustulosis, maculopapular rash, erythema nodosum, pemphigoid reactions ***Medical fact***: Dermatological side effects can appear and/or persist after the termination of the use of amoxicillin
**Ibuprofen**	***Common*** (1% to 10%): Rash, maculopapular rash, pruritus ***Very rare*** (less than 0.01%): Stevens–Johnson syndrome, erythema multiforme, toxic epidermal necrolysis ***Frequency not reported***: Ecchymosis, purpura, alopecia, sweating, photosensitivity, angioedema, exfoliative dermatitis, urticaria, vesiculobullous eruptions, Henoch–Schönlein vasculitis **For Patent Ductus Arteriosus in Pediatric Patients**: ***Very common*** (10% or more): Skin lesion/irritation (16%)
**Pantoprazole**	***Less common*** (<1%): Flushed, dry skin ***Incidence not known***: Blistering, peeling, or loosening of the skin, hives, itching, or skin rash, pale skin, red skin lesions, often with a purple center
**Contraceptive pill**	***Common*** (1% to 10%): Acne ***Uncommon*** (0.1% to 1%): Urticaria, rash, chloasma ***Rare*** (less than 0.1%): Erythema nodosum, erythema multiforme, hereditary angioedema exacerbated ***Frequency not reported***: Herpes gestationis, hirsutism, alopecia, melasma ***Post-marketing reports***: Alopecia, itching, angioedema

**Table 6:** Exemplary overview of disorders with ultrasound application and corresponding lesions (Table adapted from: Levy et al. (2021) [31].

Skin Disorder	Sonographic Changes
**Basal Cell Carcinoma**	Hypoechoic lesion with well-defined borders, usually found in dermis
**Squamous Cell Carcinoma**	Hypoechoic lesion, less apparent than BCC, harder to diagnose by ultrasonography
**Post-radiation Angiosarcoma**	Ill-defined inhomogeneous hypoechoic area, with multiple anechoic reticulated channels [34]
**Melanomas**	Homogenous hypoechoic lesion, ultrasonography having the ability to assess the thickness of the lesion
**Sclerosing disorder in atrophic skin**	Thinning of the dermis with an echogenicity similar to the unaffected surrounding skin
**Psoriasis**	Skin thickness was increased, and the echo-intensity was lower, in contrast to the adjacent skin [35]
**Hidradenitis suppurativa**	Earliest sign of follicular widening eventuating in the formation of fistulous tracts and increased vascularity over acutely inflamed lesions
**Idiopathic facial aseptic granuloma**	Poorly defined oval-shaped hypoechogenic lesion in the dermis surrounded by areas of hyperechogenicity and hypervascularization due to inflammation
**Botulinum Toxin applications**	Increased subcutaneous echogenicity leading to a blurring of the normally discrete boundaries between the subcutis and underlying muscle
**Vitamin C therapy**	Increased echogenicity in both the epidermis and dermis after 40 days and to a greater extent after 60 days

## Data Availability

The data presented in this study are available on request from the corresponding author.
